# Development of GROVE: A Guideline for RepOrting Vignette Experiments conducted in a healthcare context

**DOI:** 10.1016/j.pec.2025.108750

**Published:** 2025-03-13

**Authors:** Marij A. Hillen, Leonie N.C. Visser, Nanon H.M. Labrie, Liesbeth M. van Vliet, Som Saha, Danielle Blanch-Hartigan, Ellen M.A. Smets

**Affiliations:** a Department of Medical Psychology, Amsterdam UMC, Amsterdam, the Netherlands; b Amsterdam Public Health, Quality of Care, Amsterdam, the Netherlands; c Division of Clinical Geriatrics, Center for Alzheimer Research, Department of Neurobiology, Care Sciences and Society, Karolinska Institutet, Stockholm, Sweden; d Department of Bioethics and Health Humanities, Julius Center for Health Sciences and Primary Care, University Medical Center Utrecht, Utrecht University, Utrecht, the Netherlands; e Department of Language, Literature & Communication, Vrije Universiteit Amsterdam, the Netherlands; f Department of Pediatrics/Neonatology, OLVG Amsterdam, the Netherlands; g Amsterdam UMC, University of Amsterdam, Vrije Universiteit, Emma Children’s Hospital, Amsterdam, the Netherlands; h Leiden University, Dept of Health and Medical Psychology, the Netherlands; i Division of General Internal Medicine, Johns Hopkins University, Baltimore, USA; j Center for Health and Business, Department of Natural and Applied Sciences, Bentley University, Waltham, MA, USA

**Keywords:** Medical communication, Vignettes, Experiment, Analogue patients, Reporting guideline development

## Abstract

**Objective:**

Experimental studies using vignettes investigate the impact of healthcare professional or patient/client characteristics, communication, and/or other behaviors on outcomes. To ensure methodological rigor and quality, guidance is needed for systematic reporting of such studies. We describe the development of the Guideline for RepOrting of Vignette Experiments (GROVE).

**Methods:**

A steering group comprising experts in vignette research oversaw guideline development using an iterative and expert-driven approach. The development process included reviewing relevant literature, developing draft reporting criteria, soliciting feedback from a working group of international experts, applying the draft criteria to completed or planned vignette studies, and iteratively revising criteria until final group consensus was reached. GROVE was registered with the EQUATOR network repository of reporting guidelines.

**Results:**

The final guideline encompasses the following criteria: 1. Rationale for a vignette design; 2. Vignette content; 3. Outcomes; 4. Vignette validity & realism; 5. Participants; and 6. Accessibility. Criterion 2 is further divided into five sub-criteria: 2.1. Healthcare scenario; 2.2. Manipulation & standardization; 2.3. Mode of delivery; 2.4. Expert involvement; and 2.5. Pilot testing.

**Conclusion:**

GROVE offers authors guidance in reporting experimental vignette studies.

**Practice implications:**

Transparent reporting of vignette studies will help readers evaluate the reliability and validity of study findings, replicate studies, and extract relevant information for reviews.

## 1. Introduction

Vignettes have been used in experimental healthcare research to systematically study the effects of healthcare professional or patient behavior and/or other characteristics such as clinician attire or medical record language [1–8]. Vignettes are scripted scenarios, delivered in a written, video, audio, or extended/virtual/augmented reality format [9]. These vignettes are designed to resemble real-life situations, for example, health care consultations. Different versions of the same scenario are usually created, in which specific aspects are systematically varied while standardizing all other elements. This procedure allows researchers to experimentally compare different conditions to assess how specific factors affect outcomes, including, but not limited to, cognition (e.g., recall, understanding, test scores), affect (e.g., stress, uncertainty), evaluations (e.g., satisfaction, trust), and behavioral intentions (e.g., treatment decision-making, communication strategies) [8,10]. Vignette experiments enable researchers to make causal inferences without having to manipulate actual healthcare situations, thus providing a methodologically sound alternative to investigating effects in real life. Experimentally manipulating characteristics, communication or other behavior can be practically infeasible in real life, and is often unethical in clinical settings [10].

Participants in vignette experiments are typically assigned randomly to one or more vignette variants. They may be instructed to imagine themselves being in the position of one of the portrayed characters, or be asked to respond from a third-party perspective. If the portrayed character is a patient, participants may be referred to as ‘analogue patients’, functioning as proxies for real-life patients [11,12]. Similarly, researchers can use analogue healthcare professionals, parents, disease-naïve individuals, or care partners as research participants. After being exposed to the vignette, participants complete the outcome measures relevant to the research question at hand. In addition, psychophysiological assessments are sometimes used to provide clues about participants’ emotional and cognitive functioning while being exposed to the vignette(s) [13].

Although vignette experiments are increasingly being used in healthcare research [2,14,15], researchers are offered little guidance in how to conduct or report on these studies. This may result in variability and lack of comprehensiveness in reporting, inhibiting readers’ ability to understand the study design, evaluate the study’s reliability and validity, replicate the study, or extract relevant information for reviews. Indeed, results of two recent systematic reviews of vignette experiments within healthcare showed variations in rigor and transparency of reporting [16,17]. While more general guidelines for reporting experimental studies exist [18], these do not account for several specific methodological features of vignette experiments, particularly concerning their hypothetical nature. For example, the well-known CONSORT statement for reporting randomized trials includes general aspects such as ‘interventions’, ‘participants’ and ‘outcomes’ [18]. It does not, however, address the methodological choices researchers using vignette-based experiments face and need to justify. These may include aspects of the interventions (e.g., validity and realism of the chosen manipulations), participants (e.g., choice of analogue participants), or of the outcomes (e.g., how the selected hypothetical outcomes relate to real-world outcomes of interest). Transparency in reporting about these aspects is crucial, as there is no ‘one size fits all’ for this study design. This means that –depending on the specific study aims, context and practical limitations– various choices are made in the design and execution of vignette experiments. Readers of work reporting vignette experiments should be able to fully reconstruct such methodological choices, as these determine the balance between experimental rigor and ecological validity, among other factors. We therefore aimed to develop a reporting guideline specific to experimental studies that use vignettes in a healthcare context.

## 2. Methods

### 2.1. Design

A steering group was formed consisting of seven authors with extensive experience regarding experimental vignettes (LV, MH, NL, NV, SS, DBH, ES). These researchers had previously published and presented together on this methodology (e.g., [19]). The steering group coordinated a two-step development approach, involving expertise from a larger working group: (1) development of preliminary guideline, based on a roundtable conference session and literature search (se Sections 2.2), and (2) two feedback rounds involving experts from the working group (see Section 2.3). Throughout the development process, the steering group met online to process feedback and discuss subsequent steps.

### 2.2. Development of preliminary guideline

A 1-h openly accessible roundtable session was organized during the 2022 International Conference on Communication in Healthcare, in Glasgow (UK). Its purpose was to gather insights from known experts and interested novices on the need, structure and content of a methodological guideline for reporting on vignette experiments. A list of methodological considerations derived from a prior publication on experimental video vignettes was used to stimulate discussion [15]. Through interactive discussion, it was decided that: (1) the envisioned guideline would be separate from - rather than an extension of - the CONSORT statement or other existing guideline; (2) the focus would be on *experimental* vignettes within *health care* specifically; and (3) both the *development* and *deployment* of vignettes would be covered by the guideline. Additionally, initial thoughts were gathered on what reporting domains and criteria should be incorporated into the guideline and why. Roundtable participants who expressed interest in staying involved were included in the working group.

Next, the steering group registered the proposed guideline development by the acronym GROVE (Guideline for RepOrting Vignette Experiments) at the EQUATOR repository (May 2023). Based on referencing of Hillen et al. [10], two steering group members (LV, NL) conducted a succinct literature search of experimental vignette studies within healthcare communication, to check for any methodological issues that might have been overlooked. The search was limited to Pubmed, and conducted on 17 February 2022, using variants of the following combination of search terms: [experimental vignette] AND [communication OR behavior]. Examination of the retrieved papers yielded no newly emerging criteria. Based on existing literature, roundtable input and the literature search, the steering group established a preliminary list of reporting criteria. The preliminary guideline initially contained 15 criteria, which were reduced to 13 through continuous discussion within the steering group. We collapsed criteria that were conceptually overlapping and removed what we felt was covered by other checklists. Steering group members developed a description for each criterion, which was further refined through mutual feedback.

### 2.3. Expert feedback round

Potential working group members were identified with various expertise, to ensure broad input and uptake of the guideline. The following considerations guided the selection, in descending order of importance: (1) Expertise, i.e., evidence of methodological consideration regarding vignette experiments; (2) Experience, i.e., having conducted (preferably multiple) vignette experiments; and (3) Representation, i.e., representing various professional roles (e.g., education, editorial, statistical) and geographical areas. Experts were identified through personal networks, during the roundtable session (see 2.2), and via a search of recently published vignette experiments. In total, the working group included 15 experts. Characteristics of the full group (working and steering group) are displayed in Table 1. All working group members were involved as co-authors throughout the process of guideline creation and manuscript writing. Expertise of steering and working group members covered various topics within healthcare interaction, as well as beyond (e.g., healthcare inequality, risk behavior, and population screening).

In Round One, nine experts from the working group provided written feedback on the preliminary guideline. The feedback was processed within the steering group, resulting in a revised preliminary guideline containing 10 criteria. In Round Two, six working group members commented on the revised preliminary guideline. They were instructed to (1) apply the reporting criteria to their own recent vignette-based work and report their experiences, and (2) comment more generally on clarity, comprehensiveness, and/or redundancy of the criteria. Throughout the two feedback rounds, the most substantial changes made to the guideline included:

- Merging multiple criteria into one, or splitting one criterion into two;
- Removing any criteria or descriptions that were not specific to vignette experiments;
- Clarifying or expanding descriptions, for example by adding examples;
- Changing the criteria presentation order; and
- Changing the criteria names.

After collating the feedback from Round Two, the guideline was finalized with feedback from all working group members. The final guideline contains six criteria, with criterion two containing five sub-criteria. For more insight into previous iterations of the guideline, interested readers can contact the authors.

## 3. Results

We provide descriptions and details on the reporting criteria for vignette-based experimental studies. Table 2 presents a concise list of the GROVE criteria. A checklist for authors using the guideline is included as a supplementary file, to be completed for their specific research (see Appendix 1).

### 3.1. Rationale for use of vignettes

Explicitly state and carefully explain why an experimental vignette design was selected as the most suitable approach for the research aim (s). This may include explaining why the study could not be conducted in a naturalistic or non-simulated setting. Experimental vignette studies may represent a first step before addressing the research question in a naturalistic setting and/or allow for a controlled environment in which specific characteristics can be tested in isolation. A vignette design may be necessary because an experimental manipulation could create unacceptable risks to participants or would be practically unfeasible. For example, a study evaluating the impact of patients’ demographic characteristics and behaviors on clinicians’ decisions to prescribe opioids would not have been feasible to conduct in clinical practice while keeping all potential confounders constant [20].

### 3.2. Vignette content

Describe in as much detail as possible how the vignette content was developed and refined, and explain choices made between different options that may be consequential for the validity or reliability of the study. This will enable replicability and help readers understand the rationale for the methodological approach.

#### 3.2.1. Healthcare context

Describe and justify the healthcare context. Specify if and what sources were used to inform the script and/or other vignette content. For example, were the vignettes based on recordings of actual consultations, interviews, clinical experience, expert guidance, guidelines, patient education materials, and/or existing (theoretical) frameworks? Include information about all characters presented in the vignette, specifying their respective roles (e.g., healthcare provider, patient, caregiver, or anyone else present). Key characteristics of the portrayed characters should be specified (e.g., age, gender, ethnic identity, medical history). At a minimum, report the setting characteristics of the vignette, including medical specialty or clinical context, location, and healthcare activity or type of interaction.

#### 3.2.2. Manipulation and standardization

Experimental vignettes seek to systematically vary aspects of a healthcare context (e.g., clinician or patient appearance or communication) to test the impact of those factors on a particular outcome(s). These variations constitute ‘experimental manipulations’. For example, a researcher interested in studying the impact of non-verbal communication in a video vignette of a patient-clinician interaction could manipulate the clinician’s level of eye-contact [21]. Describe the experimental manipulations of interest, and how these were systematically varied in the vignettes. Describe the specific phenomenon or construct under investigation, then describe exactly how this was operationalized in the vignette, for example, by sharing which elements of the scenario were varied and how. Explain the number and type of manipulated independent variable(s) present in the vignettes. Include whether the manipulation occurred through the presence or absence of the independent variable or a manipulation of the number or intensity of that variable. For example, instead of saying that “smiling was manipulated,” include details about the presence or absence of smiling or the systematic variation in the number of smiles or the intensity of the smiles. The rationale, strengths and limitations of choices regarding the manipulation(s) should also be reported. For example, when manipulating symptomatology of patients presenting with post-traumatic stress disorder, the specific description of symptoms in the vignettes may influence outcomes and should therefore be reported [14]. Report how elements of the vignette other than the manipulated elements were kept constant (e.g., context, dialogue, mannerisms/non-verbal behavior, and/or length/duration). For video, audio, or virtual reality (VR) vignettes, provide information on the duration per vignette variant; for written vignettes, provide the average reading time and/or number of words or lines.

#### 3.2.3. Mode of delivery

Vignettes can be delivered in via various modalities including written, audio-only, video, and extended reality. Describe and justify the mode of delivery and provide any information necessary to replicate vignette delivery. This may include, for example, sharing the VR headset model or screen size used for delivering a video. Describe how research participants are introduced to the vignette and its context, for example, whether through oral, written or video introduction. Describe and justify the manner and setting in which the vignette is presented to participants and in which data are collected. For example, discuss the rationale and implications of delivering a vignette via an online survey in participants’ homes compared to in a research lab or clinical environment. Any potential limitations or problems encountered during vignette delivery should be discussed. If the vignette is applied in multiple languages, describe the process of translating the vignette content and context. Describe and justify choices made regarding narrative perspective and/or actors (for video or audio vignettes). For example, is the narrative voice in the first or third person? Who is the presumed narrator and why?

#### 3.2.4. Expert involvement

Discuss the involvement of relevant experts throughout vignette development process. Describe who were involved and how they contributed. Experts may include individuals with lived experience and/or relevant professional knowledge, such as patients, care partners, healthcare professionals, scholars, linguistic literary reading comprehension specialists, and educators. Explain each expert’s unique contributions throughout the process, including conceptualization, script writing, pilot-testing, translation, and other activities. Also, describe how patient perspectives were incorporated into the design process. In sum, this section should highlight the diverse expertise that contributed to the vignette development.

#### 3.2.5. Pilot testing

Describe and justify if, how, and when pilot testing was used in the vignette development process and how this affected the vignette content and format. This includes pretesting of not only the final vignette reported in the study but also any specific aspects or parts that were pre-tested during development. Report the number of pilot iterations, the characteristics and number of individuals involved in pilot testing, the basic measures/questions used as part of the pilot (e.g., realism, flow, emotional impact), key outcomes, and any revisions made to the vignette.

### 3.3. Outcomes

Outcomes in experimental vignette research by definition serve as proxies for actual outcomes. Therefore, researchers conducting vignette experiments may operationalize outcome measures and instruct participants in different ways compared to other approaches such as observational research. A broad range of outcomes is available, ranging from attitudes and affect, to hypothetical decisions, to psychophysiological measurements. Describe the study outcome(s) and explain the rationale for their selection and operationalization. Outline the measures taken to approximate reality as closely as possible.

### 3.4. Vignette validity and realism

Report how success of the experimental manipulations of interest was evaluated (i.e., manipulation check). For example, in a study testing the impact of stigmatizing vs. neutral language in patient chart notes on physician learners’ clinical decision-making, survey items were included to assess participants’ perceptions of the vignette physician’s attitudes [22]. Given the hypothetical nature of vignette-based research, reporting on validity should additionally include measures of how realistically the vignette portrays the healthcare scenario in question. Describe if and how the vignette was assessed for perceived realism and aspects of participant engagement with the healthcare scenario, using participant ratings [23–25].

### 3.5. Participants

Include a specific description of the sample participants, i.e., who is eligible to take part. Provide a rationale for the choice of study participants in relation to both the population to which findings will be generalized, and to the characters portrayed in the vignettes. While every study must consider how their participants reflect the target population, this is particularly important in vignette experiments using analogue patients. Describe if and how participants (e.g., analogue patients) were guided to become engaged with and/or identify with the scenario and its portrayed characters.

### 3.6. Availability

Given that the development process for vignettes may be extensive, dissemination of materials is recommended. Consider making the vignettes available or sharing aspects of these with other researchers (e.g., the full text, screen shots of a video, or online access to full audio vignettes). Include information regarding the availability of vignettes for research, training, educational, and/or commercial purposes. Detail any restrictions on access, whether and how other researchers are allowed to modify vignettes, and specify what uses or modifications require permissions. Indicate availability of vignette development and pilot data. Provide steps for accessing available materials.

## 4. Discussion and conclusion

### 4.1. Discussion

We described the development of the GROVE reporting guideline for vignette experiments in healthcare contexts. This method has unique methodological characteristics and challenges. Despite the powerful potential of this design to test causal relations, researchers employing experimental vignettes are often asked to justify how such research generalizes to “real world” healthcare settings. Use of the GROVE criteria will advance this type of research by enabling the research community –including researchers, editors and peer reviewers– to understand methodological choices and assess rigor and validity. Moreover, widespread uptake of the guideline will enhance replicability of studies in future research.

We recommend including the completed checklist in any publication reporting on vignette experiments in healthcare. Researchers who are using non-experimental vignette-based designs or who conduct vignette experiments outside of healthcare settings can also consider using the guideline. They should determine the applicability of each criterion for their situation. Also, researchers may find some criteria to be less relevant for their specific study. We recommend explaining in the checklist whenever this is the case. The GROVE criteria are specifically focused on *vignettes*. For guidelines regarding broader aspects of experimental or survey research, such as research background, participant recruitment, we refer to the EQUATOR database for additional reporting guidelines (www.equator-network.org) [18].

We want to emphasize the flexibility of the reporting structure we provide. The order of reporting these criteria is not intended to dictate a structure or step-by-step approach but can be flexibly presented in a manuscript. Information can be combined or reorganized, and not all information may be placed within the methods section of a manuscript. Information also does not have to be presented solely in text; figures tables, appendices, and supplementary materials may be used.

The GROVE guideline is not intended as a ‘how-to guide’, but rather as a tool to aid researchers in describing and explaining their choices. Nevertheless, the guideline may help researchers without prior experience in conducting experimental vignette research to design such a study. For more guidance on making optimal methodological choices when designing and conducting vignette experiments, other literature is available. For example, in a recent publication, Cox et al. [26] provided an overview of the empirical support substantiating methodological choices for *video* vignette experiments specifically. Evidence regarding the impact of some specific methodological choices is also available. For example, using vignette participants’ self-reported engagement, Visser et al. have reported on the differential effects of various camera perspectives, introduction formats, and vignette modalities [27,28]. King et al. reported on similarities between analogue patients with or without the health issue of concern [29]. Further methodological studies are needed to provide more empirical guidance to researchers conducting vignette experiments in making optimal methodological choices [8,15].

The GROVE guideline should not be considered as set in stone. New technological advancements are stimulating the emergence of new vignette-based methods, such as augmented reality. These might entail additional or new methodological considerations such as descriptions of technology interfaces or measures of immersion in the interaction, that are not included in the current guideline [30].

Some strengths and limitations should be noted. An important strength is our extensive and systematic approach to guideline development, which entailed a literature search and multiple feedback rounds involving a diverse group of experts and potential end users. A first limitation is that most steering group members’ experience is limited to vignette experiments focused on *clinical communication*. We may have had insufficient consideration for methodological issues pertaining to behavior or characteristics other than communication, although we have minimized this risk by including experts who have conducted other types of vignette experiments in our working group. Second, despite our broad search to identify experts with relevant expertise, we may have missed some, which potentially limits variety in viewpoints. Third, after elaborate discussion we decided not to involve patients or healthcare professionals – the stakeholders who eventually benefit from the outcomes of vignette experiments– in our development process, because of the methodological focus of the guidelines. It is possible that including these stakeholders would have yielded additional insights.

## 5. Conclusion

We created the GROVE reporting guideline for vignette experiments in healthcare through an iterative process involving 22 experts. The GROVE criteria presented here cover the conception, development and application of experimental vignettes in healthcare research. The GROVE guideline can guide researchers conducting vignette experiments, as well as reviewers and editors in assessing comprehensive methodological reporting. In this way, the guideline will help to assess and improve methodological rigor.

### Practice implications

The GROVE reporting guideline is intended to establish a standard within the research community regarding the methodological reporting of vignette experiments. This standard may ultimately contribute to improved quality and reputation of research within this field. The GROVE criteria can serve as a practical checklist for those embarking on vignette research, providing guidance for study design and reporting. Authors reporting on vignette experiments are encouraged to complete the checklist provided in the supplementary file (see Appendix 1) and include it with their manuscript. We advise that journal editors as well as peer reviewers recommend or require the use of the guideline to encourage rigor and potential replicability of vignette experiments.

## Supplementary Material

Supplemental Materials

## Figures and Tables

**Table 1:** Characteristics of steering group and working group members combined (N = 22).

Characteristic	Description	n

Gender	Female	17
	Male	5
Country of residence	The Netherlands	8
	United States of America	5
	United Kingdom	4
	Australia	2
	Germany	2
	France	1
Professional position	Full professor	6
	(Clinical/postdoctoral) Research fellow	5
	Assistant professor	5
	Junior researcher / Research assistant	3
	Associate professor	2
	Scientific officer	1
Discipline*	Psychology	11
	Psychometrics/statistics	6
	Public health / Population health	5
	Communication Science	3
	Medicine	3
Domain*	Research	22
	Teaching	15
	Clinical practice	3
	Policy	1
Experience with vignette-based research*	Involved in development of ≥ 1 study	20
	Conducted/reported on ≥ 1 study as first author	19
	Reported on ≥ 1 study as co-author	17
	Planning to conduct a study	14
Modality of vignettes used*	Written	18
	Video	15
	Audio	6
	Extended /Virtual /Augmented Reality	3

* Total number exceeds N = 22, as working group members could select multiple options.

**Table 2:** GROVE reporting criteria with descriptions*.

	Criterion	Description

**1**	**Rationale**	Provide a rationale for the use of an experimental vignette-based design, including an explanation why the study could not be conducted in a non-simulated setting.
**2**	**Vignette content**	Describe in detail how the vignette content was developed and refined, and explain any choices made.
	**2.1 Healthcare scenario**	Describe and explain in detail how the healthcare scenario was developed and what it entailed. Include information about the sources used to inform vignette content, key characteristics of the portrayed characters, and the setting described in the vignette.
	**2.2 Manipulation & standardization**	Describe what the experimental manipulations are (i.e., operationalization of the phenomenon under study), detailing which elements of the scenario were varied and how. Also report how other elements in the vignette were kept constant and provide information on vignette duration or length.
	**2.3 Mode of delivery**	Describe and explain the delivery modality and provide any information necessary for replication. Explain choices regarding narrative perspective and amount of detail described. Describe how participants were introduced to the vignette and in which setting data were collected.
	**2.4 Expert involvement**	Explain who were involved in developing the vignettes, highlighting their particular expertise and contributions.
	**2.5 Pilot testing**	Describe if, how, and when pilot testing was used in the vignette development process. Explain whether and how this affected the vignette content and format.
**3**	**Outcomes**	Explain the selected study outcome(s) for the vignette study, particularly how these outcome (s) relate to real-world outcomes of interest.
**4**	**Vignette validity & realism**	Report how manipulation success of the independent variable(s) of interest was evaluated (i.e., manipulation check). Also describe if and how realism and aspects of participant engagement with the scenario were assessed.
**5**	**Participants**	Provide a rationale for the choice of study participants (e.g., analogue patients), both in relation to the target population and to the characters portrayed in the vignettes.
**6**	**Availability**	Include information on the availability of the final vignettes and pilot data for research, teaching, or commercial purposes. Detail any restrictions to access and (re)use of the vignettes and data.

* The order of reporting these criteria is intended to be flexible. Information can be combined or reorganized and information may be placed in any manuscript section, figure, table and/or supplementary material, depending on the study content and journal requirements.

## Appendix A. Supporting information

Supplementary data associated with this article can be found in the online version at doi:10.1016/j.pec.2025.108750.
