# Supplementary material for: Development of GROVE: A Guideline for RepOrting Vignette Experiments conducted in a healthcare context
Source: Patient Educ Couns. Author manuscript; Available in PMC 2026 Mar 5. (PMC12961645; doi:10.1016/j.pec.2025.108750)
Supplement: Supplemental Materials [file NIHMS2139834-supplement-Supplemental_Materials.docx]

**Checklist form - Guideline for RepOrting Vignette Experiments (GROVE)***

Note: this document was originally published in the journal ‘Patient Education and Counseling’ under Creative Commons Attribution 4.0 International License (see <http://creativecommons.org/licenses/by/4.0/>). No changes were made to the original document, which was included as Appendix 1 to the following article: Hillen, M.A., Visser, N.C., Labrie, N.H.M. et al. Development of GROVE: a Guideline for RepOrting Vignette Experiments conducted in a healthcare context. Pat Educ Couns (2025). DOI: <https://doi.org/10.1016/j.pec.2025.108750>

| **Criterion** | **Description** | **Location in manuscript where item is reported** | **Details on methodological approach**** |
| --- | --- | --- | --- |
| **1. Rationale** | Provide a rationale for the use of an experimental vignette-based design, including an explanation why the study could not be conducted in a non-simulated setting. |  |  |
| **2. Vignette content** | Describe in detail how the vignette content was developed and refined, and explain any choices made. |  | *Report all relevant information below (sub-criteria 2.1-2.5)* |
| ***2.1. Clinical scenario*** | Describe and explain in detail how the healthcare scenario was developed and what it entailed. Include information about the sources used to inform vignette content, key characteristics of the portrayed characters, and the setting described in the vignette. |  |  |
| ***2.2. Manipulation &   standardization*** | Describe what the experimental manipulations are (i.e., operationalization of the phenomenon under study), detailing which elements of the scenario were varied and how. Also report how other elements in the vignette were kept constant and provide information on vignette duration or length. |  |  |
| ***2.3. Mode of delivery*** | Describe and explain the delivery modality and provide any information necessary for replication. Explain choices regarding narrative perspective and amount of detail described. Describe how participants were introduced to the vignette and in which setting data were collected. |  |  |
| ***2.4. Expert   involvement*** | Explain who were involved in developing the vignettes, highlighting their particular expertise and contributions. |  |  |
| ***2.5. Pilot testing*** | Describe if, how, and when pilot testing was used in the vignette development process. Explain whether and how this affected the vignette content and format. |  |  |
| **3. Outcomes &   participant   instructions** | Explain the selected study outcome(s) for the vignette study, particularly how these outcome(s) relate to real-world outcomes of interest. |  |  |
| **4. Vignette validity &   realism** | Report how manipulation success of the independent variable(s) of interest was evaluated (i.e., manipulation check). Also describe if and how realism and aspects of participant engagement with the scenario were assessed. |  |  |
| **5. Participants** | Provide a rationale for the choice of study participants (e.g., analogue patients), both in relation to the target population and to the characters portrayed in the vignettes. |  |  |
| **6. Accessibility** | Include information on the availability of the final vignettes and pilot data for research, teaching, or commercial purposes. Detail any restrictions to access and (re)use of the vignettes and data. |  |  |

*The order of reporting these criteria is intended to be flexible. Information can be combined or reorganized and information may be placed in any manuscript section, figure, table and or supplementary material, depending on the study content and journal requirements.

**Authors may report additional methodological details in this column beyond the information included in their main manuscript.
